# Effects of Acrylamide on the Activity and Structure of Human Brain Creatine Kinase

**DOI:** 10.3390/ijms10104210

**Published:** 2009-11-20

**Authors:** Qing Sheng, He-Chang Zou, Zhi-Rong Lü, Fei Zou, Yong-Doo Park, Yong-Bin Yan, Shan-Jing Yao

**Affiliations:** 1 Department of Chemical and Biochemical Engineering, Zhejiang University, Hangzhou 310027, China; 2 Yangtze Delta Region Institute of Tsinghua University, Jiaxing 314050, China; 3 Department of Environmental Health, School of Public Health and Tropical Medicine, Southern Medical University, Guangzhou 510515, China; 4 State Key Laboratory of Biomembrane and Membrane Biotechnology, Department of Biological Sciences and Biotechnology, Tsinghua University, Beijing 100084, China

**Keywords:** acrylamide, human brain creatine kinase, inactivation, docking simulation

## Abstract

Acrylamide is widely used worldwide in industry and it can also be produced by the cooking and processing of foods. It is harmful to human beings, and human brain CK (HBCK) has been proposed to be one of the important targets of acrylamide. In this research, we studied the effects of acrylamide on HBCK activity, structure and the potential binding sites. Compared to CKs from rabbit, HBCK was fully inactivated at several-fold lower concentrations of acrylamide, and exhibited distinct properties upon acrylamide-induced inactivation and structural changes. The binding sites of acrylamide were located at the cleft between the *N*- and *C*-terminal domains of CK, and Glu232 was one of the key binding residues. The effects of acrylamide on CK were proposed to be isoenzyme- and species-specific, and the underlying molecular mechanisms were discussed.

## 1. Introduction

Acrylamide, an α,β-unsaturated reactive molecule [1], is widely used worldwide in industry. For example, it is used to synthesize polyacrylamide, a material that is frequently used in laboratories for gel electrophoresis experiments [2–4]. In daily life, acrylamide can be formed during the cooking and processing of foods via the decarboxylation of the Schiff base derived from carbonyl reactants, resulting in human consumption of relatively high doses of acrylamide [2–4]. The harmful effects of acrylamide have been proposed to be caused by its neurotoxicity [5–8], genotoxicity [9], carcinogenicity [10,11], reproductive and developmental toxicities [10] and cancer risk [11]. Among these toxic effects, epidemiological studies have shown that the neurotoxicity of acrylamide affects the nerve terminal functions. Acrylamide can modify the cysteine residues of presynaptic proteins, thereby significantly reducing the neurotransmitter release, which eventually leads to process degeneration [7,12]. Particularly, creatine kinase has been found to be significantly inhibited by acrylamide in the brain or sciatic nerve according to both *in vitro* and *in vivo* studies [13–15].

**Figure 1.**
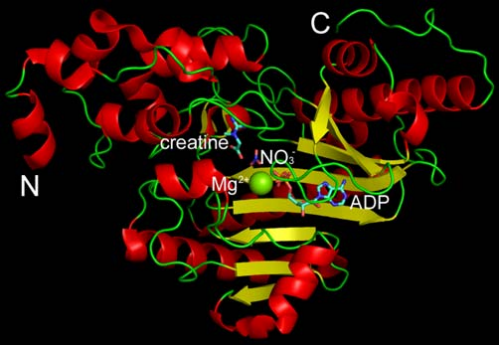
Structure of HBCK monomer (PDB ID: 3B6R) [20]. N and C denote the *N*- and *C*-terminus of the protein. The positions of the substrates are highlighted by the stick model.

Creatine kinase (CK, EC 2.7.3.2), a member of the phosphagen kinase family, catalyzes the reversible phosphotransfer between the ATP/ADP and Cr/PCr systems [16]. CK is highly expressed in vertebrate excitable tissues that require large energy fluxes and plays a crucial role in intracellular energetics [20–22]. There are four major CK isoforms, which are named according to their tissue distribution or subcellular localization [17]. Two tissue-specific (muscle or brain) cytosolic CKs exist as homo- (MM and BB) or hetero-dimers (MB) composed of muscle (M) or brain (B) monomers, while two mitochondria isoenzymes can exist as dimer or octamer [18]. CKs share a high similarity in their primary sequences and core structures (Figure 1) [19,20], while the minor difference may correlate to their isoenzyme-specific functions [21]. The ubiquitous brain-type BB-CK is widely distributed in brain, heart, smooth muscle, nervous system and other tissues, whereas the muscle-type CK (MM-CK) is the predominant isoform in highly differentiated skeletal muscle tissue [18,22]. Due to its crucial role in vertebrate energy metabolism, BB-CK is involved in many vital physiological processes and serious diseases [22–29]. CK has also long been used as a model enzyme, and its catalytic mechanism, structure, stability and folding has been extensively studied [19].

Although BB-CK has been shown to be one of the targets of acrylamide [13,14], the underlying molecular mechanism is still unclear. Previous studies suggested that acrylamide can inhibit rabbit MM-CK (RMCK) and BB-CK (RBCK) by modifying the thiol groups of cysteines [15,30]. It is worth noting that BB-CK, but not MM-CK, is the predominant CK isoform in the brain and nervous system. Moreover, previous studies have identified the isoenzyme-specific functions and stability in CKs [21,31–33]. Thus it is worthwhile to investigate the effects of acrylamide on human BB-CK (HBCK) activity and structure. In this research, we investigated the effect of acrylamide on HBCK by activity assay, spectroscopic techniques and molecular simulation studies. The results herein are expected to further our understanding of the molecular mechanism of the toxic effects of acrylamide under physiological conditions.

## 2. Results and Discussion

### 2.1. Inactivation of HBCK by Acrylamide

**Figure 2.**
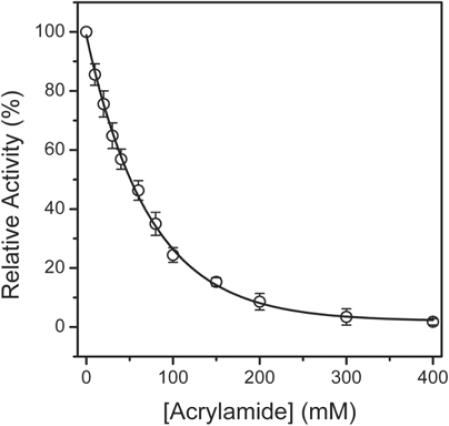
Effect of acrylamide on the activity of HBCK. The residual activity was measured after 2 h incubation of HBCK in 50 mM Tris-HCl buffer, pH 8.0, with the addition of various concentrations of acrylamide at 25 °C. The final enzyme concentration was 2 μM. The data are presented as average ± standard errors for three repetitions.

The effects of acrylamide on HBCK activity was evaluated by measuring the residual activity after incubation of the enzyme with various concentrations of acrylamide for 2 h. The data in Figure 2 clearly indicate that HBCK was inactivated by acrylamide in a dose-dependent manner. The catalytic activity of HBCK was completely eliminated by ∼300 mM acrylamide, and the *IC*_50_ value was ∼50 mM. It is worth noting that HBCK was much more easily to be inactivated by acrylamide than CKs from rabbit (RMCK and RBCK), which could still maintain about 10% of its activity at an acrylamide concentration of 600 mM and had an *IC*_50_ value of ∼200 mM [15]. Although it is not clear whether acrylamide is harmful to rabbit CKs, the results herein strongly suggested that the human BB-CK was much more sensitive to acrylamide-induced inactivation than CKs from rabbit.

**Figure 3.**
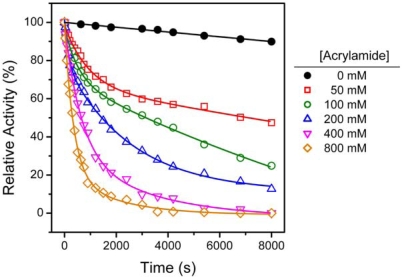
Inactivation kinetics of HBCK by various concentrations of acrylamide ranging from 0 to 800 mM. The enzyme solutions were mixed with various concentrations of acrylamide, and aliquots were taken at the indicated time points. Then the residual activity was measured using the standard activity assay, and the data were normalized by taking the activity recorded at 0 min as 100%. The data were fitted by a biphasic process, and the fitted data are presented as solid lines. The rate constants are presented in Table 1.

The inactivation kinetics were measured by time-course inactivation studies in the presence of various concentrations of acrylamide, and the results are presented in Figure 3. The time-dependent decrease of the enzyme activity of HBCK was best-fitted to a biphasic process, and the fast- (*k*_1_) and slow-phase (*k*_2_) rate constants are presented in Table 1. The changes in the transition free-energy (ΔΔ*G*°′) were also calculated by Equation 1 as described previously [30]:

(1) ΔΔGo'=−RTlnk′

where *k′* is a time constant for the major phase (fast phase) of the inactivation reaction, *T* is the absolute temperature, and R is the Boltzmann constant. These results indicated that neither the inactivation rate constants nor ΔΔ*G*°′ were significantly affected by the different concentrations of acrylamide, and the values were at the same order of magnitude.

Both RBCK and HBCK exhibited a biphasic process during acrylamide-induced inactivation, but they had different rate constants [30]. However, only a monophasic inactivation process was discovered for RMCK [15]. The difference might be caused by the relatively shorter time used for the study of RMCK or dissimilar inactivation mechanisms of the two isoenzymes by acrylamide. A comparison of the inactivation rate constants of HBCK/RBCK and RMCK indicated that although significant deviation was found for the fast phase rate constants, the slow phase rate constants of HBCK inactivation by acrylamide was almost identical for the three enzymes. It has been proposed that the inactivation of RBCK and RMCK by acrylamide was due to thiol depletion [15,30]. Because all CKs share a similar three-dimensional structure and the modification of thiol groups by acrylamide has the same rate constant, it is safe to conclude that the slow phase of HBCK inactivation was due to thiol depletion. This deduction was also supported by the fact that the rate constants of inactivation and thiol depletion were at the same level [15,30]. The fast phase inactivation of the two BB-CKs might be due to specific binding of the acrylamide molecules to the enzyme. The comparison of the inactivation processes of the three enzymes also suggested that the inactivation efficiency of acrylamide was isoenzyme- and species-dependent.

### 2.2. Effect of Acrylamide on the Tertiary Structure of HBCK

**Figure 4.**
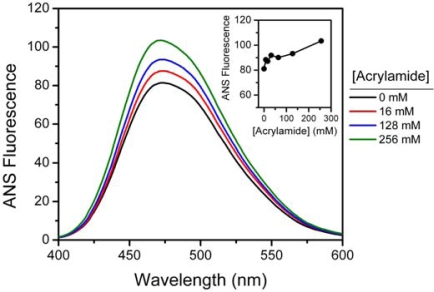
Effect of acrylamide on the ANS fluorescence of HBCK. The enzyme solutions were mixed with various concentrations of acrylamide and equilibrated for 2 h. The final concentration of ANS was 40 μM, and the solutions were incubated at ambient temperature for 30 min in the dark before measurements. The final enzyme concentration was 2 μM. The presented spectra were obtained by subtracting the spectra of ANS in the same buffer.

To elucidate the structural changes of HBCK by acrylamide, ANS extrinsic fluorescence was measured to probe the tertiary structural changes of HBCK induced by the addition of acrylamide. ANS has long been used as an extrinsic fluorescence probe to monitor the hydrophobic exposure of proteins [34]. As presented in Figure 4, the native HBCK exhibits typical ANS fluorescence emission maximum at around 470 nm, suggesting that, like MMCK [26,35–37], native BBCK molecules also contain ANS-binding sites.

In the presence of acrylamide, the intensity of ANS emission fluorescence increased slightly (∼20%) in an acrylamide concentration-dependent manner (inset of Figure 4), implying that the addition of acrylamide had a minor effect on the hydrophobic exposure of HBCK. At acrylamide concentrations above 256 mM, the ANS fluorescence had a significant blue-shift, accompanied by a decrease in intensity (data not shown), suggesting that high concentrations of acrylamide might interfere with the ANS fluorescence measurements.

It is worth noting that the tertiary structural changes of HBCK were different from those of RBCK, although they are highly homogenous in their primary sequences. Our previous study has shown that no significant change was observed at acrylamide concentrations below 800 mM for RBCK [30]. Their dissimilar structural stability coincided with their different response to acrylamide-induced inactivation.

**Figure 5.**
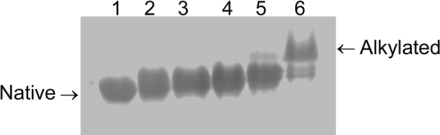
Native-PAGE analysis of the tertiary structural changes of HBCK induced by acrylamide. The protein was dissolved in 50 mM Tris-HCl buffer, pH 8.0, in the presence of various concentrations of acrylamide. Lanes 1–6 indicate the protein incubated in the buffer with the addition of 0, 50, 100, 200, 400 and 800 mM acrylamide, respectively.

Native-PAGE analysis was also performed to probe the change in the shape of the molecule (Figure 5), and the results were similar to those for RMCK and RBCK [15,30]. The shift of the band at acrylamide concentrations below 400 mM indicated that the tertiary structure of HBCK was modified by acrylamide, while the quaternary structure was not. The splitting of the band at acrylamide concentrations above 400 mM suggested that the thiol groups was alkylated by acrylamide, as proposed previously [30].

### 2.3. Acrylamide Binding-Site on HBCK by Docking Simulation

**Figure 6.**
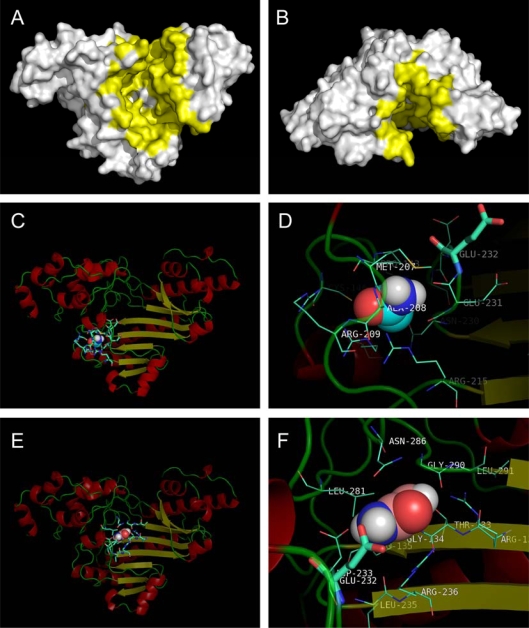
The surface of HBCK (A and B) and acrylamide binding sites predicted by Autodock (C and D) and Fred (E and F). (A and B) The surface of the HBCK molecule. The yellow parts indicated the position of the cleft or pocket between the two domains of HBCK. Panel (B) shows the top view of the structure shown in panel (A). (C-F) The residues forming the binding site are shown by a line model, the acrylamide molecule is presented by a space-filling model, while Glu232 is highlighted by a stick model.

The above results from inactivation and structural studies suggested that acrylamide might have specific binding sites on HBCK. Actually, a previous study suggested that the acrylamide binding-site at RBCK was at a pocket around Cys74 according to docking simulation of a predicted RBCK structure [30]. Recently, the crystal structures of HBCK in ligand-free and ligand-binding forms have been published [20], and the results provided reliable structures for docking simulation studies. As presented in Figure 1, each subunit of CK contains two domains: a smaller *N*-terminal domain and a larger *C*-terminal domain, and the active site of CK is located at the cleft of the two domains [19]. This cleft or pocket is thought to facilitate the entry of substrates as well as inhibitors [19,30]. The HBCK pocket, which is estimated to be as large as ∼2,500 Å^2^, is shown in Figures 6A and 6B.

Three HBCK structures have been reported: the ligand-free (PDB ID: 3DRE), ADP-binding (PDB ID: 3DRB) and TSAC-binding (PDB ID: 3B6R) forms. To ensure the reliability of the docking results, all the crystal structures were used for the docking simulations, and no significant difference in the binding sites were found for the three crystal structures. The docking was also performed by both Autodock 4.0 and Fred 2.0 software programs, and the docking of the acrylamide molecule to the HBCK molecule was successful as indicated by the statistically significant scores (about −3.7 kcal/mol for Autodock and about −30 kcal/mol for Fred 2.0). Furthermore, we also tested the reliability by docking ADP to the ligand-free HBCK molecule, and the RMSD was 0.214 Å, which confirmed that the docking results were reliable.

Surprisingly, the two programs give different sites of acrylamide-binding (Figure 6). Considering that the weak binding of acrylamide and different CK isoforms had dissimilar *IC*_50_ values, it is possible that CK contains multiple acrylamide binding sites. Moreover, the biphasic inactivation process also suggested that BB-CK might have acryamide binding sites with distinct binding affinities. This could also explain why different sites were predicted by the two programs and why different sites were predicted for the two highly homologous BB-CK enzymes.

The binding site predicted by Autodock was formed by residues Pro143, Cys146, Arg151, Met207, Ala208, Arg209, Arg215, Asn230, Glu231, Glu232, Asp233 and His234, while that by Fred was formed by residues Thr133, Gly134, Arg135, Glu232, Asp233, Leu235, Arg236, Leu281, Asn286 and Gly 290. These two potential acrylamide binding sites were adjacent, and two residues (Glu232 and Asp233) were predicted to participate in acrylamide binding for both cases. Previous structural and mutational studies have pointed out that Glu232 is one of the crucial residues responsible for CK catalysis by participating in the positioning of creatine (Figure 1) [19,20,30]. Thus the binding of acrylamide might impair the structure and flexibility of the active site by altering the position of Glu232, resulting in HBCK inactivation.

A comparison of the results obtained herein and those of RMCK and RBCK [15,30] strongly suggested that the inhibition effect of acrylamide on CK might be isoenzyme- and species-specific. The structure and sequence are highly conserved among all CKs [19], and the identity in the primary sequence is 96.6% between HBCK and RBCK, and 80.1% between HBCK and RMCK. For all CKs, there is a large pocket between the *N*- and *C*-terminal domains to facilitate the entry of the substrates. Both the docking simulations in this research and that for RBCK suggested that the acrylamide binding sites were located at this pocket, but at different positions. These observations implied that acrylamide might have multi-binding sites on CK, while the affinity and position might influenced the structure of the active site in different ways. The disruption of the active site conformation might allow the modification of the thiol group of Cys283, a fully conserved residue crucial for CK, and further inactivate the enzyme. This proposal is consistent with the distinct IC50 values observed for the three CK isoenzymes, and could very well explain why different acrylamide binding sites were obtained for HBCK and RBCK by the two simulation programs. The species- and isoenzyme-specific response of CK against acrylamide-induced inactivation also suggested that great caution should be taken when using model proteins from other species to evaluate the effects of toxicants on human beings.

## 3. Experimental Section

### 3.1. Materials and Protein Expression and Purification

Creatine, ATP, DTT, magnesium acetate, thymol blue, acrylamide, and ANS were all purchased from Sigma. All the other reagents were local products of analytical grade. Recombinant HBCK was expressed and purified as described previously [30,37]. HBCK purity was confirmed by the presence of only one band in both SDS- and native-PAGE analyses, as well as LC-MS/MS identification. The protein concentration was determined according to the Bradford method by using bovine serum albumin as a standard [38].

### 3.2. HBCK Activity Assay

HBCK activity was measured according to the pH-colorimetry method by following the proton generation during the reaction of ATP and creatine [39]. The enzymatic activity was monitored by the absorption at 597 nm with an Ultraspec 4300 pro UV/Visible spectrophotometer (Amersham Pharmacia Biotech), and the indicator was thymol blue. The reaction and measurements were performed at 25 °C. The assay buffer contained 24 mM creatine, 4 mM ATP, 8 mM magnesium acetate, 0.01% thymol blue, and 5 mM glycine-NaOH (pH 9.0).

### 3.3. ANS Fluorescence

The extrinsic fluorescence emission spectra were collected using a Hitachi F-2500 spectrofluorimeter (Tokyo, Japan) using 1-cm-pathlength cuvettes. The protein solutions with various amounts of acrylamide were incubated with 40 μM ANS for 30 min in the dark. An excitation wavelength of 390 nm was used for ANS-binding fluorescence, and the emission wavelength ranged from 400 to 600 nm.

### 3.4. In silico Docking of HBCK and Acrylamide

To ensure the reliability of the docking results, all of the three published HBCK crystal structures (PDB IDs: 3DRE, 3DRB and 3B6R) [20] were used for the *in silico* docking simulations. Two programs, Autodock 4.0 and Fred 2.0, which are based on different search techniques, were used in this research [40,41]. The original structure of acrylamide was obtained from the PubChem database (http://www.pubchem.org; compound ID: 6579) [42]. Details regarding the procedures of docking simulations were the same as those described previously [30,43]. The reliability of the simulations was also examined by docking the ADP molecule to the ligand-free HBCK molecule, and the RMSD was 0.214 Å, which confirmed that the docking results were reliable.

## 4. Conclusions

Acrylamide, which is widely used worldwide in industry and can be produced by the cooking and processing of foods, has been found to be harmful to human beings. BB-CK has been proposed to be one of the important targets in the neurotoxicity of acrylamide. In this research, we studied the effects of acrylamide on human BB-CK activity, structure and the potential binding sites. Compared to CKs from rabbit, HBCK was fully inhibited at several-fold lower concentrations of acrylamide, and exhibited distinct properties upon acrylamide-induced inactivation and structural changes. The highly conserved structure of CKs but quite dissimilar responses suggested that the effects of acrylamide might be isoenzyme- and species-specific. The binding sites of acrylamide were proposed to be located at the cleft between the N- and C-terminal domains of CK, and the affinities of the multiple sites might be accounted for the slight difference in the sequence and structure of various CKs. This might be the structural basis of the different effect of acrylamide on various CKs. The results herein provide insight into the inhibitory effects and the possible ligand-binding mechanism of acrylamide on CK.

## Figures and Tables

**Table 1.** Inactivation rate constants of HBCK in the presence of acrylamide.

**Acrylamide (mM)**	**Inactivation rate constants (10^−3^ s^−1^)**		**ΔΔ*G*°′ (kJ/mol·min^−1^)**
	***k*_1_**	***k*_2_**	
50	2.0	0.31	25.5
100	2.9	0.21	24.6
200	5.7	0.39	22.9
400	3.5	0.65	24.1
800	3.6	0.68	24.0
